# Cost Utility Analysis of Internet-Based Cognitive Behavioral Therapy for Major Depressive Disorder: Randomized Controlled Trial

**DOI:** 10.2196/67567

**Published:** 2025-02-19

**Authors:** Wenjing Zhou, Yan Chen, Herui Wu, Hao Zhao, Yanzhi Li, Guangduoji Shi, Wanxin Wang, Yifeng Liu, Yuhua Liao, Huimin Zhang, Caihong Gao, Jiejing Hao, Gia Han Le, Roger S McIntyre, Xue Han, Ciyong Lu

**Affiliations:** 1 Department of Medical Statistics and Epidemiology School of Public Health Sun Yat-sen University Guangzhou China; 2 Guangdong Provincial Key Laboratory of Food, Nutrition and Health Sun Yat-sen University Guangzhou China; 3 Department of Psychiatry Shenzhen Nanshan Center for Chronic Disease Control Shenzhen China; 4 Brain and Cognition Discovery Foundation Toronto, ON Canada; 5 Institute of Medical Science University of Toronto Toronto, ON Canada; 6 Mood Disorder Psychopharmacology University Health Network Toronto, ON Canada; 7 Department of Psychiatry University of Toronto Toronto, ON Canada

**Keywords:** cost utility analysis, CUA, cost-effectiveness, economic evaluation, costs, quality of life, internet-based cognitive behavioral therapy, ICBT, digital psychiatry, major depressive disorder, depression, China

## Abstract

**Background:**

Unguided internet-based cognitive behavioral therapy (ICBT) has been proven effective for major depressive disorder (MDD). However, few studies have examined its cost-effectiveness in low-resource countries and under nonspecialist routine care.

**Objective:**

This study aimed to evaluate the short- and long-term cost utility of unguided ICBT compared to a waitlist control for persons with MDD from the perspectives of society and the health care system.

**Methods:**

This analysis was implemented alongside an 8-week 2-arm randomized controlled trial with a 12-month follow-up period conducted in Shenzhen, China. Outcomes including cost and health utility were collected at the pretreatment and posttreatment time points and 3, 6, and 12 months after the intervention. Direct medical costs and indirect costs were prospectively collected using the hospital information system and the Sheehan Disability Scale. Health outcomes were measured using the Chinese version of the Short-Form Six-Dimension health index. The primary outcome was incremental cost utility ratio (ICUR) expressed as the difference in costs between 2 therapies by the difference in quality-adjusted life years (QALYs). The seemingly unrelated regression model and the bootstrap method were performed to estimate adjusted ICURs. Cost-effectiveness planes and cost-effectiveness acceptability curves were used to demonstrate uncertainty. A series of scenario analyses were conducted to verify the robustness of base-case results.

**Results:**

In total, 244 participants with MDD were randomly allocated to the ICBT (n=122, 50%) or waitlist control (n=122, 50%) groups. At the pretreatment time point, no statistically significant difference was observed in direct medical cost (*P*=.41), indirect cost (*P*=.10), or health utility (*P*=.11) between the 2 groups. In the base-case analysis, the ICBT group reported higher direct medical costs and better quality of life but lower total costs at the posttreatment time point. The adjusted ICURs at the posttreatment time point were CN ¥–194,720.38 (US $–26,551.50; 95% CI CN ¥–198,766.78 to CN ¥–190,673.98 [US $–27,103.20 to US $–25,999.70]) and CN ¥49,700.33 (US $6776.99; 95% CI CN ¥46,626.34-CN ¥52,774.31 [US $6357.83-$7196.15]) per QALY from the societal and health care system perspectives, respectively, with a probability of unguided ICBT being cost-effective of 75.93% and 54.4%, respectively, if the willingness to pay was set at 1 time the per-capita gross domestic product. In the scenario analyses, the probabilities increased to 76.85% and 77.61%, respectively, indicating the potential of ICBT to be cost-effective over the long term.

**Conclusions:**

Unguided ICBT is a cost-effective treatment for MDD. This intervention not only helps patients with MDD improve clinically but also generates societal savings. These findings provide health economic evidence for a potential scalable MDD treatment method in low- and middle-income countries.

**Trial Registration:**

Chinese Clinical Trial Registry (ChiCTR) ChiCTR2100046425; https://tinyurl.com/bdcrj4zv

## Introduction

### Background

Major depressive disorder (MDD) is a highly prevalent mental disorder estimated to affect 332 million people worldwide in 2021 [1]. MDD is associated with significant impairment of health-related quality of life (HRQoL) [2], substantial health care use [3], and huge societal impacts on employment and productivity [4,5]. Now, MDD is ranked as the second leading cause of global nonfatal disability [1], and MDD-attributed lost productivity costs the global economy US $1 trillion annually [6]. Effective, scalable, and cost-effective therapies are needed to reduce the disease burden caused by MDD [7].

Despite the availability of effective first-line treatments involving psychotherapies and pharmacotherapies for MDD [8], the treatment gap is still large [9,10]. Individuals with MDD are faced with a series of barriers to treatment, including social discrimination, stigma, health care costs, and a shortage of specialists [11,12], especially in low-resource settings such as China [13]. Unguided internet-based cognitive behavioral therapy (ICBT), a form of cognitive behavioral therapy delivered through the internet without therapeutic support [14], has the attributes of privacy, accessibility, flexibility, and ease of implementation with constrained health care resources and personnel [15,16]. It has been gradually applied for the treatment of MDD. Existing research across low-, middle-, and high-income countries has demonstrated the effectiveness of unguided ICBT for MDD in comparison with usual care [11,17-20]. In China, our team has also developed an unguided ICBT course for MDD, and its efficacy has been confirmed through a randomized controlled trial (RCT) [21].

In contrast with the well-established evidence of effectiveness, the cost-effectiveness of unguided ICBT for MDD remains relatively under-studied [22-30]. There is an increasing demand for economic evidence to optimize resource allocation and scale up the implementation of unguided ICBT in different contexts [31]. However, existing trial-based economic evaluations are mostly conducted in high-income countries and adopt a single perspective (societal or health care system perspective), and the results are inconclusive [32-34]. Previous cost utility analyses (CUAs) conducted from the perspective of the health care system over a long-term follow-up period (1-2 years) have reported that unguided ICBT had lower probability of being more cost-effective in comparison with usual care at setting values of willingness to pay (WTP), with the probability of unguided ICBT being cost-effective ranging from 3.8% to 41.7% [26-28,30]. In the case of those adopting the societal perspective, the findings have been mixed, and the supportive evidence has had some uncertainties [22-25,29,30]. Holst et al [30] conducted a CUA of unguided ICBT compared with usual care among participants with mild to moderate depression in Sweden and found that no firm conclusions could be drawn under a range of assumed WTP. In the Netherlands, all CUA analyses have reported positive results and suggested the economic merits of unguided ICBT compared to usual care, with the likelihood of the former being cost-effective being 52%, 65%, and 62% [23-25]. In addition, another 2 evaluations conducted in the United Kingdom and Spain demonstrated that unguided ICBT was the dominant treatment option in comparison with usual care [22,29]. Considering the large heterogeneity in current research, the generalizability and external validity of the cost-effectiveness conclusions require more research in different settings under economic guidance. To our knowledge, no study has reported the cost-effectiveness of unguided ICBT for MDD in China. It is necessary to extend the analysis to China and account for both the societal and health care system perspectives to provide decision-making evidence for the optimization and allocation of the limited health care resources.

### Objectives

Therefore, this study aimed to comprehensively assess the short- and long-term cost utility of unguided ICBT among persons with MDD in China from the perspectives of society and the health care system.

## Methods

### Study Design

This economic evaluation was conducted prospectively alongside an 8-week pragmatic, unblinded, 2-arm RCT with a follow-up period of 12 months and was conducted from the perspectives of society and the Chinese health care system. The time horizon was in line with that of the RCT from baseline to 12 months after treatment. Participants were randomly allocated to the ICBT (ICBT plus usual care) or waitlist control (usual care) groups at a ratio of 1:1. The detailed study design and procedures have been described in a published paper [21].

### Ethical Considerations

This RCT was registered with the Chinese Clinical Trial Registry (ChiCTR2100046425) and obtained ethics approval from the Ethics Review Committee of Shenzhen Nanshan Center for Chronic Disease Control (ll20210012) in line with the standards stipulated in the Declaration of Helsinki. The CONSORT-EHEALTH (Consolidated Standards of Reporting Trials of Electronic and Mobile Health Applications and Online Telehealth) checklist was used to guide and report the RCT [35]. In addition, the Consolidated Health Economic Evaluation Reporting Standards were applied to conduct and guide the economic evaluation [36]. All participants provided written informed consent, including for the use of their data for secondary analyses. The data used for the analyses were deidentified to protect the privacy of the participants. For those who completed the trial, financial incentives in the form of small amounts of electronic cash (CN ¥50 [US $6.82]) were distributed.

### Study Participants

In this study, participants were recruited from the Department of Depressive Disorder, Shenzhen Kangning Hospital, and the Department of Psychiatry, Shenzhen Nanshan Center for Chronic Disease Control, between August 2021 and December 2022. To be eligible for this study, participants had to meet the following inclusion criteria: (1) age 18 to 60 years; (2) a positive screening result on the Patient Health Questionnaire–9 (PHQ-9) of ≥5 [37]; (3) a diagnosis of MDD through the Mini-International Neuropsychiatric Interview [38] through the *Diagnostic and Statistical Manual of Mental Disorders, Fourth Edition*; (4) access to internet-connected mobile devices; (5) lack of any other psychotherapy or physical therapy currently; (6) no changes in condition during the previous month (eg, patients with antidepressant use did not change the dosage); and (7) informed consent. The exclusion criteria were (1) neurological illness (eg, traumatic brain injury or functional impairment); (2) a moderate to high risk of suicide; (3) alcohol abuse or substance use disorder; (4) pregnancy or breastfeeding; and (5) diagnosis of a severe physical disease, psychosis, or bipolar disorder. Eligible participants were asked to complete the baseline assessment, including sociodemographic information (eg, age, sex, and ethnicity), clinical history (eg, antidepressant use and history of psychopathology), lifestyle (eg, smoking and drinking), and baseline clinical symptoms (measured using the PHQ-9, Generalized Anxiety Disorder–7, and Kessler Psychological Distress Scale).

### Intervention

Participants in the ICBT group were provided with an ICBT course for 8 weeks in addition to usual care. Each participant was assigned an account number and corresponding password after the pretreatment instruction and training for use. The ICBT course is called Morning Mood; is embedded in the WeChat mini program; and can be accessed via smartphones, tablets, and computers. The course was grounded in cognitive behavioral therapy principles aiming at teaching emotional regulation skills and cognitive restructuring. In total, there are seven 30-minute modules. The detailed contents and corresponding screenshots are shown in Multimedia Appendix 1. Participants were required to complete 1 module per week. Technical support and reminder services via telephone call and SMS text messaging were provided by trained nonspecialists (ie, lay health workers, nurses, and social workers) throughout the intervention and follow-up periods to promote participant engagement. However, no therapeutic guidance was offered to them, and thus, this treatment was defined as an unguided ICBT course.

Participants in the waitlist control group were on a waiting list for ICBT and did not receive any additional specific interventions. However, individuals’ usual treatment was maintained during the 8-week control period. After 8 weeks, the participants in the control arm completed the observation phase and began to receive the ICBT treatment.

### Health Outcomes

The primary health outcome was quality-adjusted life years (QALYs), estimated using the Simplified Chinese version of the Short-Form Six-Dimension (SF-6D) health index, which is demonstrated to have good validity among the Chinese population, including individuals with mental health problems [39,40]. The SF-6D is one of the most widely used preference-based HRQoL instruments [41] and has been recommended by many national guidelines of economic evaluations for the calculation of QALYs [42]. The SF-6D defines the health state through the following 6 dimensions: physical functioning, role limitation, social functioning, pain, mental health, and vitality [43]. The SF-6D responses were converted to health utility on the QALY scale from 0 (death) to 1 (full health) using the Chinese value set for the SF-6D [44], with a value of <0 indicating a worse health condition than death. The SF-6D was administered at baseline; the posttreatment time point; and the 3-, 6-, and 12-month follow-ups. Subsequently, QALYs were derived by multiplying time in a particular health state by its health utility using the area under the curve method [45], with 1 QALY indicating 1 life year in perfect health. The adjusted QALY for CUA was calculated using the regression model mentioned in later sections.

### Cost Analyses

Costs were calculated from the societal and health care system perspectives using individual-level data collected for the following periods: 3 months before randomization, from randomization to the posttreatment time point (3 months), from the posttreatment time point to the 3-month follow-up, from the 3- to the 6-month follow-up, and from the 6- to the 12-month follow-up. In total, 3 main categories of costs were identified: intervention costs, health care costs, and societal costs attributed to lost or declined productivity. The health care system perspective accounts for all the costs directly associated with health care services [46], and thus, intervention costs and health care costs are included within this perspective. As the societal perspective is the broadest perspective comprising all health care–related costs and productivity costs [46], all 3 costs are considered within this perspective.

Intervention costs refer to the expenses associated with the intervention administered. For the ICBT group, the intervention costs comprised the costs of development and maintenance of Morning Mood, implementation support, and personnel training. On the basis of the practical operation, we estimated that the costs were CN ¥545 (US $74.31) per participant. For the waitlist control group, the intervention costs were CN ¥0 per participant.

Health care costs, also called direct medical costs, comprise expenses generated from health care resource use, including but not limited to diagnostic tests, examinations, medications, and other treatments. In this study, direct medical costs were retrieved from the Shenzhen Hospital information system at the individual patient level and calculated using the bottom-up method [47].

Societal costs, also known as indirect costs, are the quantified economic value of lost and declined productivity attributed to MDD. The Sheehan Disability Scale (SDS) was applied to measure days of lost and declined productivity through the following items: “On how many days in the last week did your symptoms cause you to miss school or work or leave you unable to carry out your normal daily responsibilities” and “On how many days in the last week did you feel so impaired by your symptoms, that even though you went to school or work, your productivity was reduced” [48]. Previous research has converted absenteeism and presenteeism into monetary units in populations with mental health conditions [49]. Thus, this study also applied the human capital approach for the estimation [50]. Specifically, loss of productivity was calculated using days of lost productivity multiplied by the monthly average wage in Shenzhen [51]. For declined productivity, a weighting coefficient of 42.98% was used for the adjustment [49].

All costs were collected alongside the RCT and were measured in 2022 Chinese yuan. The baseline costs were identified as costs incurred during the 3 months before the individuals’ time of enrollment and were used to control for baseline differences.

### Statistical Analysis

Baseline characteristics and outcome measures were described by group and by phase with continuous variables using mean, SD, and frequency for categorical variables. Mann-Whitney *U* tests, 2-tailed *t* tests, and chi-square tests were used to investigate the differences in baseline characteristics and outcomes between the 2 arms. Estimates of the difference between groups in health utility scores were derived at each time point with 95% CIs and *P* values. Statistical significance was determined when the *P* value was <.05.

Missing data were imputed using the multiple imputation by chained equations package in R (version 4.2.2; R Foundation for Statistical Computing) under the assumption of data being missing at random [52,53]. There is compelling evidence that multiple imputation and bootstrapping are robust resampling approaches for dealing with skewed and missing data in cost-effectiveness trials [54]. The predictive mean matching method in multiple imputation by chained equations was adopted for imputation, which matched the missing value to the observed value with the closest predicted mean, with the advantage of preserving data distribution and avoiding values lying outside the bounds for each variable [55]. A total of 5 imputed datasets were generated and used for analysis as previous studies have suggested that 3 to 5 imputed datasets were sufficient to provide adequate estimates [56]. The rules by Rubin [57] were used to pool the results, involving the estimates, SEs, and 95% CIs. Health utility and costs at the posttreatment time point and every follow-up time point were imputed based on age, gender, antidepressant use, baseline values (utility and cost), and values from the previous or next measurement time point [26].

### Economic Evaluation

The CUA was conducted from the perspectives of society and the health care system for 8 weeks, 3 months, 6 months, and 12 months, not accounting for the discount rate for cost and QALYs. Both the complete-case (CC) and intention-to-treat (ITT) samples were considered. Seemingly unrelated regression was conducted to estimate the total costs, QALYs, and corresponding differences (incremental differences in costs and QALYs between the 2 arms) while adjusting for baseline values and other covariates and considering the correlation between costs and QALYs, with the coefficients of the treatment group (reference: waitlist control group) in 2 separate regression models representing the cost and effect differences [58]. Total costs were adjusted for baseline costs, age, gender, and antidepressant use [59]. QALYs were adjusted for baseline utility, age, gender, and antidepressant use [59,60]. Incremental cost utility ratios (ICURs) were calculated using the incremental difference in costs divided by the incremental difference in QALYs between the 2 arms. Nonparametric bootstrapping methods were performed to account for the uncertainty surrounding the estimates of cost, QALYs, and ICURs. Cost-effectiveness planes and cost-effectiveness acceptability curves (CEACs) were applied to demonstrate the uncertainty of the results [61]. In this study, the WTP threshold (ie, the amount that people are willing to pay for 1 year lived in full health state) was set at 1.5 times the per-capita gross domestic product per QALY [62], with a range from 1 to 3 times the per-capita gross domestic product (CN ¥80,976 to CN ¥242,928 [US $11,041.60 to $33,124.90]) according to the Chinese guideline for health economics [63]. Standard decision rules were used to determine whether the ICBT was cost-effective compared to the waitlist control [58].

Base-case cost-effectiveness analysis was conducted using data collected at the posttreatment time point (ie, 8 weeks). Sensitivity analyses were implemented through a series of scenario analyses using data collected at the follow-up time points. The aforementioned analyses also assessed whether the results remained consistent after adjusting for baseline differences and adopting different analysis datasets. To explore the long-term cost utility of ICBT, extrapolation analyses across various time horizons (3-, 6-, and 12-month follow-ups) for the waitlist control group were conducted as no follow-up data were available for that group. For each time point, data extrapolation for the control group was applied based on the predicted model formulated from cost and utility data in the ICBT group. Detailed operations and practical rationality have been clearly described in a published article [64]. All analyses were conducted using R (version 4.2.2) and Stata (version 17.0; StataCorp).

## Results

### Overview

**Figure 1:**
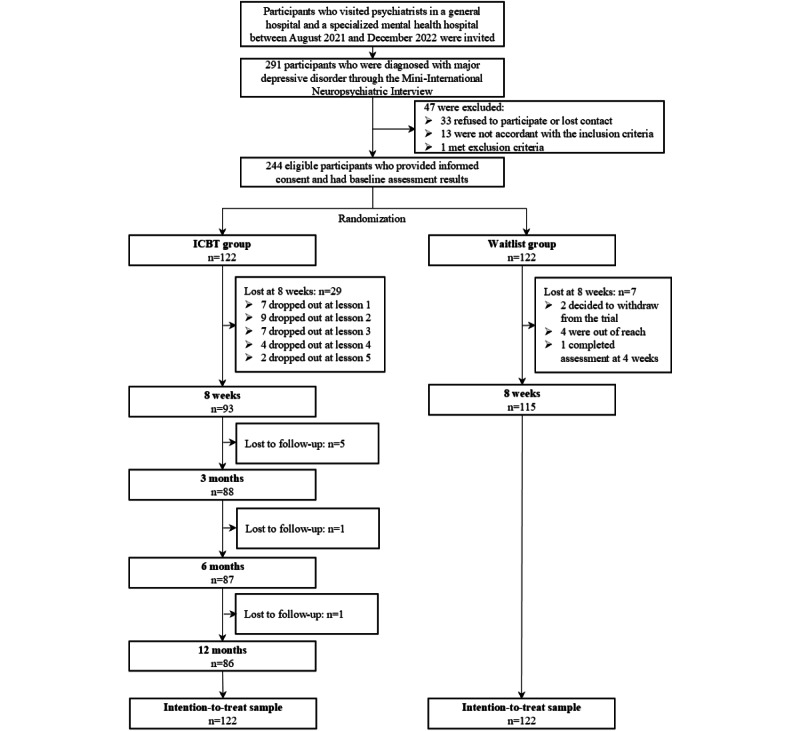
Flowchart of participants. ICBT: internet-based cognitive behavioral therapy.

Between August 2021 and December 2022, a total of 291 persons with MDD were recruited. Of these 291 participants, 33 (11.3%) either refused to participate or lost contact, 13 (4.5%) did not meet the inclusion criteria, and 1 (0.3%) met the exclusion criteria. Finally, a total of 244 eligible participants with baseline information and who provided informed consent were enrolled, with 122 (50%) participants allocated to each group. In the ICBT group, a downward trend was observed in completion rates, with the highest rate at 8 weeks (93/122, 76.2%) and the lowest rate at the 12-month follow-up (86/122, 70.5%). For the waitlist control group, a high completion rate was reported, with 94.3% (115/122) of the participants completing the posttreatment assessment. A detailed account of the study population and participant flow is shown in Figure 1.

### Baseline Characteristics

Table 1 shows the baseline characteristics of the participants by treatment group. The mean age was 28.3 (SD 7.0) years, and the female-to-male participant ratio was 3:1. Most of the sample (224/240, 93.3%) was of Han ethnicity. Most of the participants were well educated, with 82.2% (199/242) having received education above the undergraduate level. A total of 69.8% (169/242) of the participants were employed. More than half of the participants were experiencing their first episode of MDD (144/239, 60.3%) and were not receiving antidepressant treatment (127/244, 52%). The mean scores on the PHQ-9, Generalized Anxiety Disorder–7, SDS, and Kessler Psychological Distress Scale at baseline were 13.9 (SD 5.2), 10.5 (SD 4.7), 17.7 (SD 7.0), and 29.7 (SD 9.0), respectively. There were substantial differences in baseline characteristics between the ICBT and waitlist control groups except for the SDS scale scores. The mean SDS scores in the ICBT group were significantly higher than those in the control group (18.8, SD 7.0 vs 16.7, SD 6.8; *P*=.02), indicating that participants in the ICBT group had worse social functioning.

**Table 1:** Baseline characteristics of the participants (N=244).

Variable			Total		ICBT^a^ (n=122)		Waitlist (n=122)		*P* value^b^
Age (y)^c^, mean (SD)			28.3 (7.0)		28.6 (6.8)		28.0 (7.3)		.50
**Sex^c^** **, n (%)**									.14
	Female	183 (75)		86 (70.5)		97 (79.5)			
	Male	61 (25)		36 (29.5)		25 (20.5)			
**Ethnicity, n (%)**									.82
	Han	224 (93.3)^d^		112 (94.1)^e^		112 (92.6)^f^			
	Others	16 (6.7)^d^		7 (5.9)^e^		9 (7.4)^f^			
**Educational level, n (%)**									.18
	High school or lower	43 (17.8)^g^		27 (22.3)^f^		16 (13.2)^f^			
	Undergraduate	168 (69.4)^g^		80 (66.1)^f^		88 (72.7)^f^			
	Master’s degree or higher	31 (12.8)^g^		14 (11.6)^f^		17 (14)^f^			
**Current employment status, n (%)**									.11
	Student	40 (16.5)^g^		14 (11.6)^f^		26 (21.5)^f^			
	Employed	169 (69.8)^g^		90 (74.4)^f^		79 (65.3)^f^			
	Unemployed	33 (13.6)^g^		17 (14)^f^		16 (13.2)^f^			
**Recurrent episodes, n (%)**									.49
	Yes	95 (39.7)^h^		50 (42.4)^i^		45 (37.2)^f^			
	No	144 (60.3)^h^		68 (57.6)^i^		76 (62.8)^f^			
**Current drinking status, n (%)**									.16
	Yes	204 (84.6)^j^		106 (88.3)^k^		98 (81)^f^			
	No	37 (15.4)^j^		14 (11.7)^k^		23 (19)^f^			
**Current smoking status, n (%)**									.43
	Yes	101 (42.1)^d^		47 (39.2)^k^		54 (45)^k^			
	No	139 (57.9)^d^		73 (60.8)^k^		66 (55)^k^			
**Antidepressant use^c^, n (%)**									.44
	Yes	117 (48)		55 (45.1)		62 (50.8)			
	No	127 (52)		67 (54.9)		60 (49.2)			
PHQ-9^l^ score at baseline^c^, mean (SD)			13.9 (5.2)		14.2 (5.5)		13.6 (4.9)		.39
GAD-7^m^ score at baseline^c^, mean (SD)			10.5 (4.7)		10.8 (4.8)		10.2 (4.6)		.30
SDS^n^ score at baseline^c^, mean (SD)			17.7 (7.0)		18.8 (7.0)		16.7 (6.8)		.02
K-10^o^ score at baseline^c^, mean (SD)			29.7 (9.0)		29.3 (10.0)		30.1 (8.0)		.47

^a^ICBT: internet-based cognitive behavioral therapy.

^b^Baseline characteristics were compared between the 2 groups using 2-tailed *t* tests for continuous variables and chi-square tests for categorical variables.

^c^Variables with no missing data.

^d^n=240.

^e^n=119.

^f^n=121.

^g^n=242.

^h^n=239.

^i^n=118.

^j^n=241.

^k^n=120.

^l^PHQ-9: Patient Health Questionnaire–9.

^m^GAD-7: Generalized Anxiety Disorder–7.

^n^SDS: Sheehan Disability Scale.

^o^K-10: Kessler Psychological Distress Scale.

### Health Outcome

Table 2 provides the completion rates of the SF-6D scale and the converted health utility scores. At baseline, the mean utility of the ICBT group was lower than that of the control group (0.5190, SD 0.2286 vs 0.5625, SD 0.1949); however, the difference was not statistically significant (*P*=.11). After the 8-week intervention, increases in the average utility scores were observed in both groups (0.6002, SD 0.2285 vs 0.6012, SD 0.2149; *P*=.98), with completion rates of 73% (89/122) and 94.3% (115/122), respectively. The ICBT group demonstrated continuous improvement in health utility scores over the 6-month follow-up period, with a slight decline at 12 months. The highest health utility score of 0.6240 in the ICBT group was observed at the 6-month follow-up.

Multimedia Appendix 2 shows the health utility scores and subsequent QALY estimations in the ITT sample. In the ICBT group, the mean QALY gains were 0.1414 (SE 0.0021), 0.2952 (SE 0.0041), 0.4539 (SE 0.0059), and 0.7765 (SE 0.0096) at the posttreatment time point (ie, 8 weeks) and the 3-, 6-, and 12-month follow-up time points, respectively. In the control group, the average QALYs at the same time points were 0.1463 (SE 0.0019), 0.3000 (SE 0.0038), 0.4598 (SE 0.0053), and 0.7839 (SE 0.0080), respectively. There was no significant difference in QALYs between groups at any time point. Both the CC and ITT analyses suggested sustained improvement in health utility in the ICBT group over 12 months and in the waitlist control group over 8 weeks.

**Table 2:** Health utility measured using the Short-Form Six-Dimension health index by time point in the complete-case analysis.

Health utility at different time points	ICBT^a^ (n=122)						Waitlist (n=122)							Mean difference between the 2 groups (*P* value)^b^
	Time point (T_i_)		Difference between time points (T_i_ – T_i__–__1_)			Time point (T_i_)				Difference between time points (T_i_ – T_i__–__1_)				
	Participants, n (%)	Scores, mean (SD)	Participants, n (%)	Scores, mean (SD)	Participants, n (%)			Scores, mean (SD)	Participants, n (%)		Scores, mean (SD)			
T_0_^c^	122 (100)	0.5190 (0.2286)	—^d^	—	122 (100)			0.5625 (0.1949)	—		—	−0.0435 (.11)		
T_1_^e^	89 (73)	0.6002 (0.2285)	89 (73)	0.0892 (0.1843)	115 (94.3)			0.6012 (0.2149)	115 (94.3)		0.0420 (0.1602)	−0.0010 (.98)		
T_2_^f^	87 (71.3)	0.6057 (0.2416)	83 (68)	0.0018 (0.1509)	—			—	—		—	—		
T_3_^g^	85 (69.7)	0.6240 (0.2198)	84 (68.9)	0.0172 (0.2074)	—			—	—		—	—		
T_4_^h^	82 (67.2)	0.6146 (0.2590)	81 (66.4)	−0.0088 (0.2012)	—			—	—		—	—		

^a^ICBT: internet-based cognitive behavioral therapy.

^b^Health utility at different time points was compared between the 2 groups using *t* tests.

^c^T_0_: pretreatment time point.

^d^No applicable data.

^e^T_1_: posttreatment time point (ie, 8 weeks).

^f^T_2_: 3-month follow-up.

^g^T_3_: 6-month follow-up.

^h^T_4_: 12-month follow-up.

### Costs

Table 3 shows the costs of different components and aggregated costs in the CC analysis by trial arm. At all time points, indirect costs were the major contributor to the total costs. The cost of antidepressants constituted the primary component of direct medical costs. At the pretreatment time point (ie, baseline), the average direct medical costs in the ICBT and control groups were CN ¥6588.70 (US $898.42; SD CN ¥14,093.70 [US $1921.77]) and CN ¥5861.70 (US $799.28; SD CN ¥10,198.60 [US $1390.65]), respectively. Indirect costs approximately amounted to CN ¥22,225.90 (US $3030.66; SD CN ¥16,225.30 [US $2212.43]) in the ICBT group and CN ¥18,853.10 (US $2570.75; SD CN ¥16,197.50 [US $2208.64]) in the control group. However, both lacked statistical significance. After the 8-week intervention, both the direct medical costs and indirect costs decreased in both groups. The ICBT group reported statistically significantly higher indirect costs than those in the waitlist control group (*P*=.01).

Multimedia Appendix 3 shows the results of the cost analysis in the ITT sample. Both groups revealed a decreasing trend in direct medical costs and indirect costs during the posttreatment and follow-up periods. For patients in the ICBT group, the declining trend was sustained throughout the follow-up period.

**Table 3:** Average costs per participant (in Chinese yuan) by trial arm for the complete-case analysis at the pre- and posttreatment time points.

Time point		ICBT^b^ (n=122)			Waitlist (n=122)			*P* value^a^
		Resource users, n (%)	Costs (CN ¥), mean (SD)	Resource users, n (%)		Costs (CN ¥), mean (SD)		
**Pretreatment**								
	Drug costs	64 (52.5)	5881.50 (US $801.98; 13,772.60 [US $1877.99])	65 (53.3)		4858.60 (US $662.50; 9619.10 [US $1311.63])	.68	
	Treatment costs	12 (9.8)	179.60 (US $24.49; 674.40 [US $91.96])	9 (7.4)		246.1 (US $33.56; 1365 [US $186.13])	.50	
	Diagnostic and examination costs	64 (52.5)	527.60 (US $71.94; 746.60 [US $101.8])	70 (57.4)		757 (US $103.22; 907.8 [US $123.78])	.08	
	Direct medical costs	79 (64.8)	6588.70 (US $898.42; 14,093.70 [US $1921.77])	82 (67.2)		5861.7 (US $799.28; 10,198.6 [US $1390.65])	.41	
	Indirect costs	109 (89.3)	22,225.90 (US $3030.66; 16,225.30 [US $2212.43])	107 (87.7)		18,853.1 (US $2570.75; 16,197.5 [US $2208.64])	.10	
**Posttreatment**								
	Drug costs	16 (13.1)	2281.80 (US $311.14; 11,545.30 [US $1574.28])	23 (18.9)		1336.8 (US $182.28; 4567 [US $622.74])	.24	
	Treatment costs	2 (1.6)	92.90 (US $12.67; 940.50 [US $128.24])	2 (1.6)		21.5 (US $2.93; 175 [US $23.86])	>.99	
	Diagnostic and examination costs	10 (8.2)	46.60 (US $6.35; 186.80 [US $25.47])	14 (11.5)		58.4 (US $7.96; 201 [US $27.41])	.40	
	Direct medical costs	18 (14.8)	2421.20 (US $330.15; 12,416.40 [US $1693.06])	24 (19.7)		1416.7 (US $193.18; 4697.8 [US $640.58])	.31	
	Indirect costs	109 (89.3)	14,817.30 (US $2020.44; 10,816.90 [US $1474.96])	87 (71.3)		11,115.6 (US $1515.69; 11,147.5 [US $1520.04])	.01	

^a^Costs at different time points were compared between the 2 groups using Mann-Whitney *U* tests.

^b^ICBT: internet-based cognitive behavioral therapy.

### Economic Evaluations

The adjusted mean costs and QALYs (bootstrapped SEs and 95% CIs) by seemingly unrelated regression in base-case analysis and scenario analyses for this health economic evaluation from the perspectives of society and the health care system in the CC and ITT samples are shown in Multimedia Appendices 4 and 5. In most scenarios, the adjusted costs and QALYs were not statistically significantly different between the 2 groups. The corresponding results of the incremental cost-effectiveness analysis are shown in Table 4.

**Table 4:** Summary of incremental cost-effectiveness (CE) results for the baseline-adjusted (BA) complete-case, intention-to-treat, and scenario analyses from the societal and health care system perspectives.^a^

Analysis				Difference in mean costs^b^ (bSE^c^)		Difference in mean QALYs^b,d^ (bSE)		ICUR^e^		ICURs by CE plane quadrant (%)													Probability of CE^f^ (%)		
										Southeast quadrant		Southwest quadrant		Northeast quadrant		Northwest quadrant		South quadrant		East quadrant		≤1 × GDP^g^ per capita^h^			≥3 × GDP per capita^i^
**Societal perspective**																									
	**Complete-case analysis: 8 weeks**																								
		BA cost/BA QALY	CN ¥–149.65 (US $−20.41; CN ¥1645.32 [US $224.35])		0.0042 (0.0028)		CN– ¥35,456.18 (US $–4834.70)		50.7		3.58		42.25		3.47		54.28		92.95		62.28			74.63	
	**Intention-to-treat analysis: 8 weeks**																								
		BA cost/BA QALY	CN ¥–899.45 (US $−122.65; CN ¥2064.49 [US $281.51])		0.0046 (0.0028)		CN ¥–194,720.38 (US $–26,551.50)		65.82		3.28		29.84		1.06		69.1		95.66		75.93			86.24	
	**Intention-to-treat analysis: 3 months**																								
		BA cost/BA QALY	CN ¥–1278.93 (US $−174.39; CN ¥3378.79 [US $460.72])		0.0126 (0.0079)		CN ¥–101,283.17 (US $–13,810.67)		63.36		3.03		31.88		1.73		66.39		95.24		76.85			88.46	
	**Intention-to-treat analysis: 6 months**																								
		BA cost/BA QALY	CN ¥–1761.24 (US $−244.25; CN ¥4952.93 [US $675.37])		0.0179 (0.0116)		CN ¥–98,146.80 (US $–13,383.00)		62.53		3.17		31.78		2.52		65.7		94.31		75.32			86.32	
	**Intention-to-treat analysis: 12 months**																								
		BA cost/BA QALY	CN ¥–2698.94 (US $−368.02; CN ¥7396.60 [US $1008.58])		0.0278 (0.0211)		CN ¥–97,064.62 (US $–13,235.44)		61.13		4.81		30.42		3.64		65.94		91.55		75.62			85.48	
**Health care system perspective**																									
	**Complete-case analysis: 8 weeks**																								
		BA cost/BA QALY	CN ¥507.13 (US $69.15; CN ¥161.95 [US $22.08])		0.0043 (0.0028)		CN ¥118,884.70 (US $16,210.76)		0.11		0		93.05		6.84		0.11		93.16		28.6			76.9	
	**Intention-to-treat analysis: 8 weeks**																								
		BA cost/BA QALY	CN ¥201.90 (US $27.53; CN ¥1568.36 [US $213.86])		0.0041 (0.0027)		CN ¥49,700.33 (US $6776.99)		41.75		3.02		52.22		3		44.78		93.97		54.4			71.03	
	**Intention-to-treat analysis: 3 months**																								
		BA cost/BA QALY	CN ¥136.60 (US $18.63; CN ¥1963.81 [US $267.78])		0.0110 (0.0073)		CN ¥12,400.87 (US $1690.95)		44.1		3.42		49.6		2.88		47.52		93.7		66.97			86.28	
	**Intention-to-treat analysis: 6 months**																								
		BA cost/BA QALY	CN ¥112.37 (US $15.32; CN ¥2060.10 [US $280.91])		0.0153 (0.0108)		CN ¥7332.67 (US $999.86)		44.39		3.95		48.16		3.5		48.34		92.55		72.33			88.34	
	**Intention-to-treat analysis: 12 months**																								
		BA cost/BA QALY	CN ¥68.40 (US $9.33; CN ¥2210.68 [US $301.44])		0.0228 (0.0189)		CN ¥3001.31 (US $409.25)		43.43		6.1		45.56		4.91		49.53		88.99		77.61			87.58	

^a^The scenario analysis represents a scenario whereby the waitlist control participants’ health-related quality of life and care costs after 8 weeks up to 12 months followed the same trend observed in the intervention group; therefore, the difference in costs and quality-adjusted life years is based on the observed values for the intervention group at each time point, but predicted values were obtained using regression analysis for the waitlist control group, the regression model for which is described in the Methods section.

^b^Difference in mean values between trial arms.

^c^bSE: bootstrapped SE.

^d^QALY: quality-adjusted life year.

^e^ICUR: incremental cost utility ratio.

^f^Probability of being cost-effective.

^g^GDP: gross domestic product.

^h^Willingness to pay 1 time the per-capita GDP.

^i^Willingness to pay 3 times the per-capita GDP.

**Figure 2:**
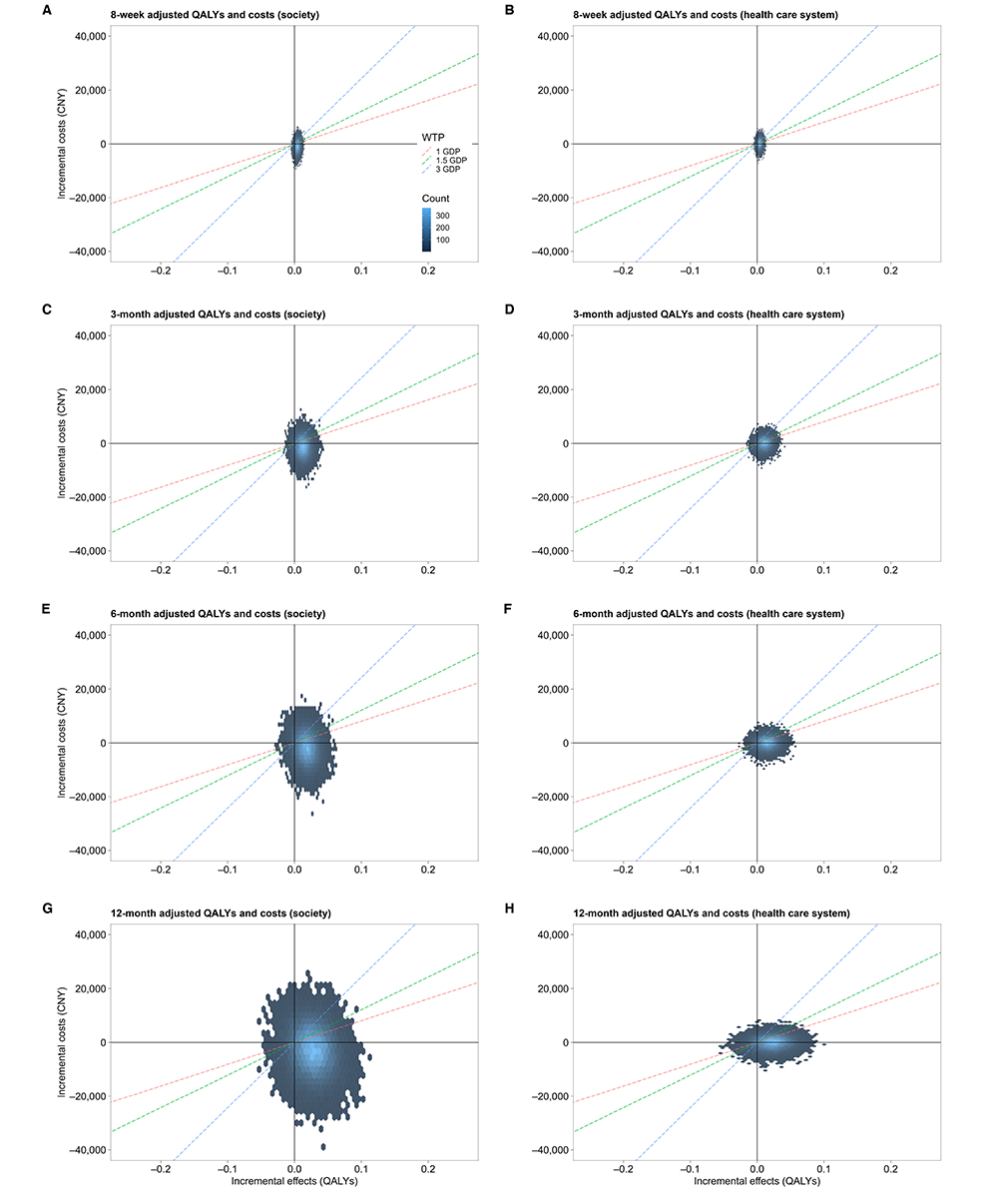
Cost-effectiveness planes showing the difference in baseline-adjusted incremental quality-adjusted life years (QALYs; x-axis) and incremental costs (y-axis) of 5000 bootstrapping samples between trial arms across 8 weeks and 3, 6, and 12 months. (A,C,E,G): society; (B,D,F,H): health care system. CNY: Chinese yuan; GDP: gross domestic product; WTP: willingness to pay.

**Figure 3:**
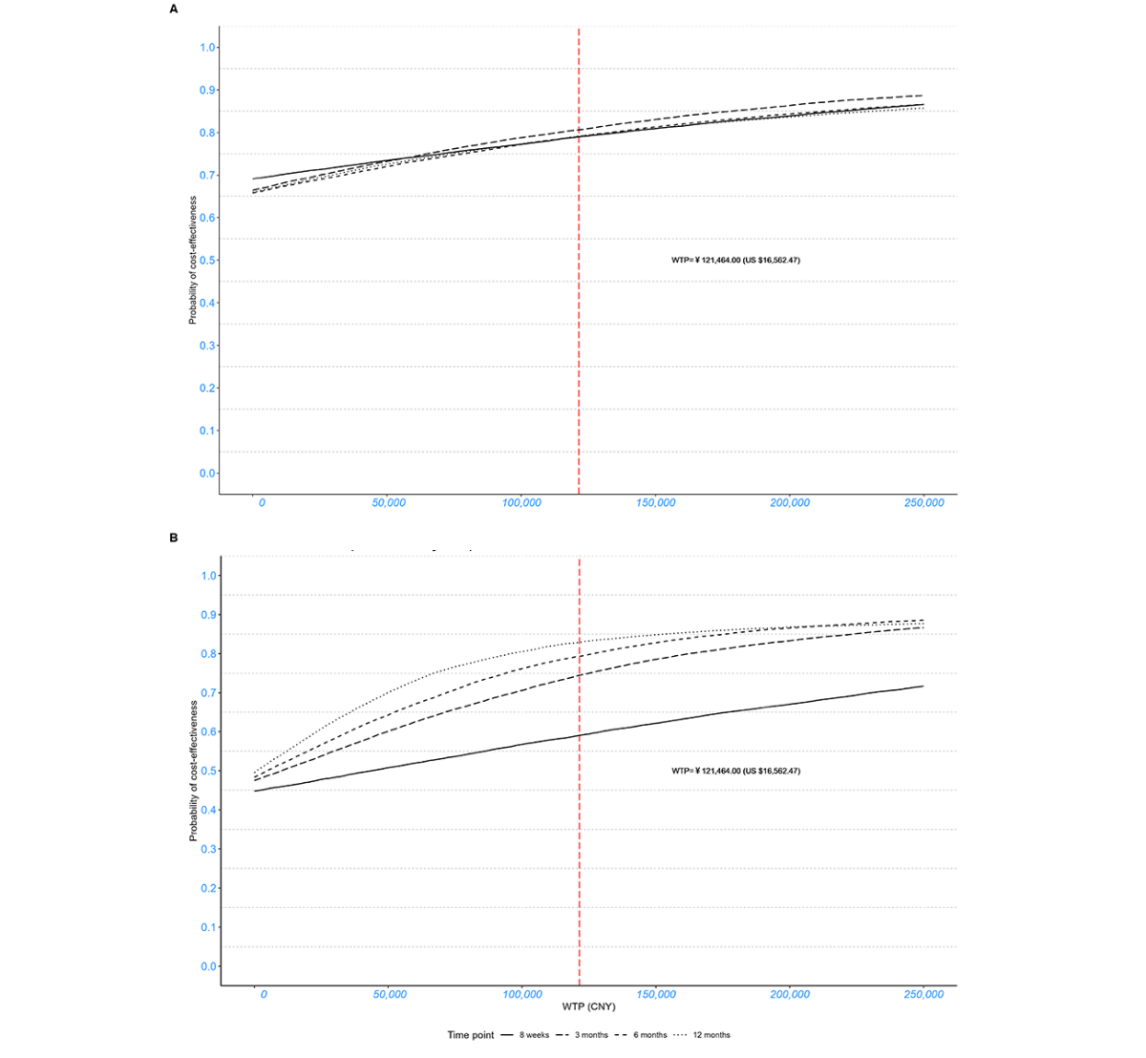
Cost-effectiveness acceptability curves representing the probability of cost-effectiveness of internet-based cognitive behavioral therapy (ICBT) relative to the waitlist control over 8 weeks and 3, 6, and 12 months (baseline-adjusted). (A) ICBT versus waitlist (society); (B) ICBT versus waitlist (health care system). CNY: Chinese yuan; WTP: willingness to pay.

From the perspective of society, the adjusted incremental costs (CN ¥−899.45 [US $–122.65]) and QALYs (0.0046) resulted in an ICUR of CN ¥−194,720.38 (US $–26,551.50) per QALY in the base-case analysis, which was much lower than the setting WTP threshold. This dominant ICUR indicates that ICBT is more cost-effective compared to the waitlist control from the societal perspective. The cost-effectiveness plane and CEAC, generated based on 5000 bootstrapping replications, further illustrated the robustness of the point estimates. Specifically, 29.84% of replications fell within the northeast quadrant (more expensive and more effective), and 65.82% fell within the southeast quadrant (less expensive and more effective; Figure 2). The probability of ICBT being cost-effective at the setting WTP ranged from 75.93% to 86.24% and showed an upward trend with the increase in WTP (Figure 3).

Similarly, analysis from the perspective of the health care system indicated that ICBT was more cost-effective than the waitlist control, with an ICUR of CN ¥49,700.33 (US $6776.99) per QALY and probabilities of ICBT being cost-effective ranging from 54.4% to 71.03%. Notwithstanding the aforementioned observation, no cost-saving effect for the health care system was observed.

### Sensitivity Analysis

Sensitivity analyses were conducted by varying follow-up duration (3-, 6-, and 12-month follow-ups) through subsequent scenario analyses. Figures 2 and 3 show the cost-effectiveness planes and CEACs, respectively, at different time points from the societal and health care system perspectives for comparison. From the societal perspective, the results similarly indicated lower costs but higher QALYs for participants in the ICBT group, with an 88.46% probability of ICBT being cost-effective, suggesting the long-term health and economic value of ICBT. In addition, from the health care system perspective, no cost-saving effect was observed. Higher direct medical costs and QALYs were observed in the ICBT group, with the probability of ICBT being cost-effective reaching 88.34%.

**Figure 4:**
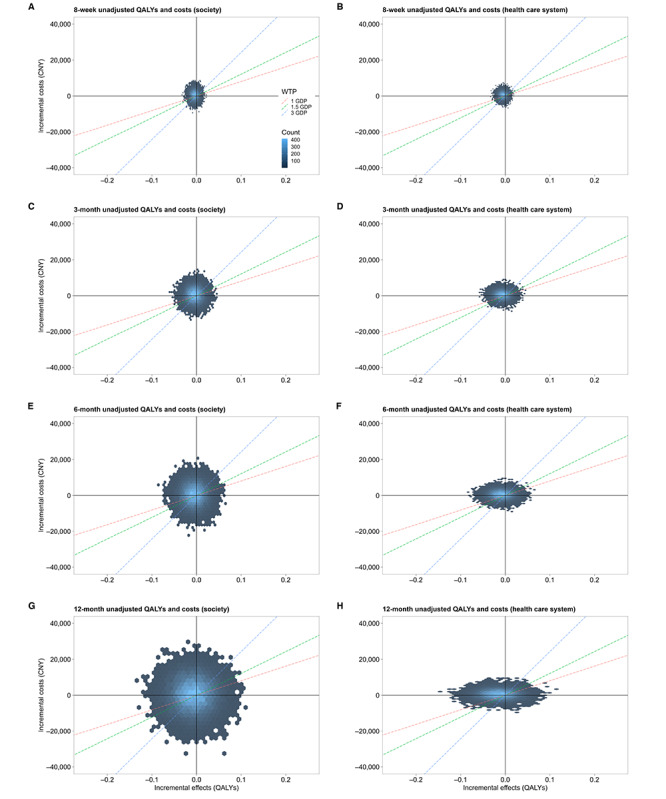
Cost-effectiveness planes showing the difference in unadjusted incremental quality-adjusted life years (QALYs; x-axis) and incremental costs (y-axis) of 5000 bootstrapping samples between trial arms across 8 weeks and 3, 6, and 12 months. (A,C,E,G): society; (B,D,F,H): health care system. CNY: Chinese yuan; GDP: gross domestic product; WTP: willingness to pay.

**Figure 5:**
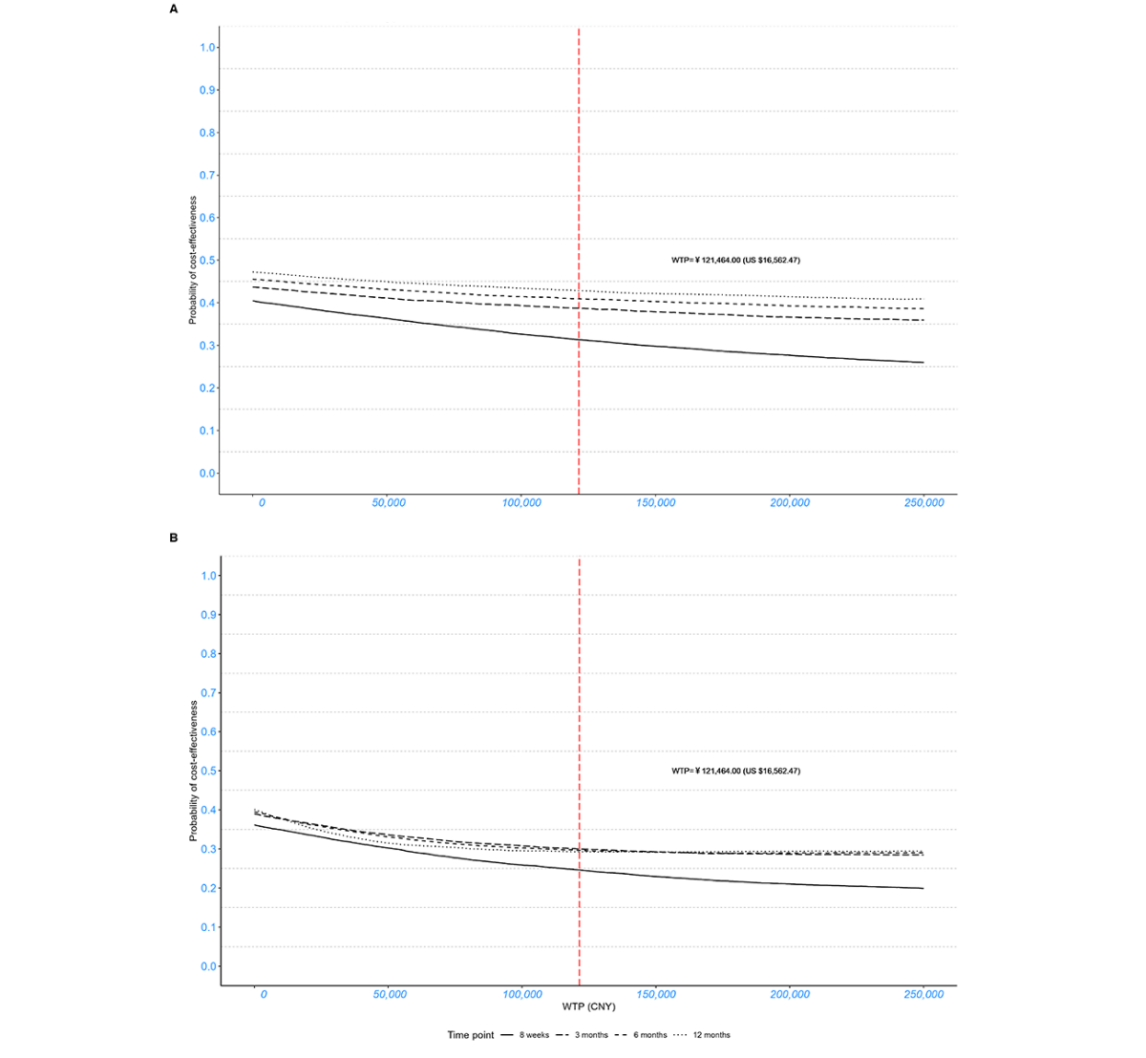
Cost-effectiveness acceptability curves representing the probability of cost-effectiveness of internet-based cognitive behavioral therapy (ICBT) relative to the waitlist control over 8 weeks and 3, 6, and 12 months (unadjusted). A) ICBT versus waitlist (society); (B) ICBT versus waitlist (health care system). CNY: Chinese yuan; WTP: willingness to pay.

When the baseline values were not adjusted, the ICBT and waitlist control groups showed no difference in terms of cost utility at all time points (Figures 4 and 5). The economic evaluation is reported following the Consolidated Health Economic Evaluation Reporting Standards 2022 checklist, which is presented in Multimedia Appendix 6.

## Discussion

### Principal Findings

This study aimed to evaluate the short- and long-term cost-effectiveness of unguided ICBT compared to a waitlist control for MDD in China. The base-case findings of this CUA showed that the ICBT intervention was more cost-effective for persons with MDD compared to the waitlist control over 8 weeks from the perspectives of society and the health care system. Sensitivity analyses further confirmed the results by changing the data analysis set (ie, CC and ITT samples), study period (ie, 3-, 6-, and 12-month follow-ups), and whether baseline conditions were adjusted for. To the best of our knowledge, this is the first cost-effectiveness study on unguided ICBT for MDD in China, contributing to the research gap in low- and middle-income countries and providing economic evidence for policy makers to rationally allocate limited health resources.

### Previous Studies

From the perspective of society, the incremental cost per QALY gain was CN ¥−194,720.38 (US $–26,551.50), which implied that the ICBT intervention was associated with higher improvement in HRQoL at lower costs, with the probability of ICBT being cost-effective ranging from 75.32% to 88.46% at the setting WTP thresholds. These findings were consistent with those of most previous research conducted in high-income countries demonstrating the cost utility of unguided ICBT for MDD [22-25,29]. Among them, one study also suggested the clinical and cost-saving effect of unguided ICBT [29], with other studies reporting more effectiveness at higher costs [22-25]. This might be owing to the different depression severity in the recruited study participants. It has been indicated that unguided ICBT is more suitable for mild depression [17]. Individuals with milder depressive symptoms are likely to benefit more from ICBT treatment, with greater responsiveness and more substantial health improvements. As a result, their social functions improve, enabling them to engage in daily activities and return to work and leading to decreased societal costs. However, a study with a small sample size conducted in Sweden reported that no firm conclusions could be drawn on the cost-effectiveness of unguided ICBT [30].

From the health care system perspective, the findings that unguided ICBT was more cost-effective than usual care (ICUR: CN ¥49,700.33 [US $6776.99]; range of probability of ICBT being cost-effective at the given WTP 54.4%-88.34%) were contrary to those of most previous cost-effectiveness studies [26-28,30]. The following different settings might explain the discrepancy: the different instruments used for the measurement of HRQoL (EQ-5D in previous studies and SF-6D in this study), varied baseline symptom severity (PHQ-9≥10 in previous studies and PHQ-9≥5 in this study), and diverse health care use patterns in different health care systems. It is assumed that persons with mild symptoms are more likely to benefit from unguided ICBT [17]. In addition, the SF-6D is more sensitive to changes in mental health, and thus, minor improvements could be captured [65]. Previous research has reported that the conclusions on cost-effectiveness were the opposite when QALYs were estimated using the EQ-5D-3L or the SF-6D [26,28]. When using the SF-6D, ICBT appeared to dominate usual care (lower mean costs and higher QALYs), whereas in our study, ICBT exhibited greater health effects at higher costs. A possible explanation could be the variations in mental health care–seeking behaviors in different contexts. In China, individuals are less likely to approach mental health care due to stigma [66]. However, the ICBT course helps people with MDD transform their inappropriate or negative thought patterns to promote health care use [67]. Hence, the costs of health care in the ICBT group appeared higher than those of the waitlist control group in our analysis. This, in turn, explains the results from the societal perspective. It is plausible that increased patient investment in mental health care and service yields improved social functioning. Consequently, reduced days of lost or decreased productivity and indirect costs associated with cost savings were observed [22,68]. It is worth mentioning that these results should be interpreted with caution and need to be confirmed by further powered studies in different contexts.

The robustness of the results of the base-case analysis was verified through different sensitivity analyses. Scenario analyses over the 3-, 6-, and 12-month follow-ups all suggested higher likelihoods of ICBT being cost-effective, aligning with previous studies and confirming the long-term cost-effectiveness of ICBT [22-25,29]. It is important to note that adjustment for differences in baseline costs and QALYs leads to slightly different results compared to those obtained without baseline correction. At lower levels of WTP, ICBT showed an even chance of being cost-effective in both baseline-adjusted and nonadjusted analyses. However, with the increase in WTP, the opposite conclusion was drawn in the choice between the 2 treatments in terms of cost utility. One plausible explanation is that the effect of the intervention on utility was mild and the differences between the intervention and control group in costs and QALYs were relatively minor [28]. It was assumed that the baseline utility and costs were strongly correlated with the subsequent estimates, potentially leading to different outcomes when not adjusting for baseline variables [24]. It was argued that, in the economic evaluation, controlling for baseline utility and costs should be necessary [60].

Despite the positive outcomes of the economic evaluation, the results suggest that there was no statistically significant difference in HRQoL (subsequent QALYs) and cost estimates between the 2 groups at most time points, which is consistent with the findings of previous economic evaluations [23-25,29,69]. It has been reported that the between-group (ICBT vs control) effect size of HRQoL is small and a longer duration is required to capture meaningful observations [17-19,70]. In this study, the sample size was calculated based on detecting the medium effect size on depressive symptoms between the 2 groups, and thus, the statistical power might not be sufficient to detect statistical differences in HRQoL. Moreover, a systematic review aimed at addressing the effectiveness of ICBT on HRQoL also found that adults with more severe depressive symptoms were more likely to achieve greater HRQoL improvements and therapeutic guidance could further enhance the effect on HRQoL [71]. Hence, this lack of statistically significant differences in HRQoL and cost estimates between the 2 groups might be partly explained by the mild symptoms (mean PHQ-9 score 13.9, SD 5.2) experienced by the participants and the unguided ICBT intervention in this study. Another possible explanation was that the measurement tool for HRQoL lacked sensitivity to minor changes in this patient group [28,72]. Regarding costs, an economic evaluation of unguided ICBT conducted among 1013 participants with mild to moderate depression severity suggested that the total costs at 6 months after enrollment did not significantly differ between the intervention and control groups [69]. Given the high variability of costs [73], research with larger sample sizes and longer time frames may be needed to detect statistically significant differences.

There were several strengths to this study. First, this economic evaluation was carried out and reported following international economic evaluation guidelines. Moreover, the outcome data were collected alongside a pragmatic RCT. Therefore, the validity and reliability of the data were guaranteed. Second, in the recording of direct medical costs, the hospital information system was used to collect data at an individual level. Hence, recall bias was reduced to some extent compared with self-reported data. In addition, the self-developed ICBT courses were embedded in the WeChat mini program, one of the most popular platforms among the Chinese population, which led to a high retention rate. Under the support of nonspecialists, the retention rate in the waitlist control group was also maintained at a high level. Thus, the changes in clinical effectiveness and quality of life might reflect the real effect of the intervention. Fourth, the implementation of our intervention was executed by nonprofessional personnel, such as primary health care workers, which was in line with practical operations in routine care, indicating that the results had good features of scalability in the current medical environment.

### Limitations

Nonetheless, this study has some limitations. The primary limitation was the short-term follow-up period in the control group due to ethical considerations. Consequently, given the time-dependent nature of QALYs, the potential lag effect of ICBT, and the results reported in previous studies, rigorous statistical extrapolations on costs and QALYs were made for the control group with the assumption that the trends in costs and QALYs were the same in the intervention and control arms. Scenario analysis was conducted based on predicated data, suggesting that the long-term cost-effectiveness results should be interpreted with caution. It is likely that ICBT is more cost-effective in a longer follow-up period. Further studies under longer time frames extending the follow-up periods or adopting strong modeling assumptions would be useful. Second, although the sample size in this RCT had sufficient statistical power to detect clinically significant improvements in depression, it did not necessarily have the statistical power to detect differences in cost and utility due to the high variability of costs and the small effect of ICBT on HRQoL. Replications with large sample sizes are needed to explore differences in costs and cost utility. In addition, identifying individuals who benefit from the ICBT course and those who are at risk of not responding and adopting tailored interventions is likely to be an important way to improve the cost-effectiveness of ICBT. However, these results need to be confirmed by more research in different subsamples. Third, as direct nonmedical cost data were unavailable, only direct medical and indirect costs were included in this analysis. The aforementioned limitation might potentially underestimate the overall total economic burden and affect the conclusions. In future studies of economic evaluations on ICBT, a comprehensive assessment of the economic burden of participants will be of significance. Fourth, in this study, female participants accounted for 75% (183/244) of the total participants, and most participants (199/242, 82.2%) were well educated, which affects the extrapolation at the population level. Future interested researchers may need to take account of the representativeness of the samples. Fifth, this study only included participants who had access to digital devices and, thus, might have excluded socioeconomically marginal populations and could raise ethical issues [74,75]. It is not clear whether addressing digital divides and ensuring equitable access (eg, providing loaner electronic devices and internet connection) would influence the extant results. This remains open for further exploration. Sixth, despite the existing evidence on effectiveness and cost-effectiveness, the use of personal data, including data privacy and participant autonomy, should be considered before the massive scale-up of ICBT [76,77].

### Conclusions

In comparison with the waitlist control, unguided ICBT is more cost-effective for MDD from both the health care system and societal perspectives in China. This intervention not only helps patients with MDD improve clinically but also generates societal savings. These findings suggest that unguided ICBT has broad application prospects in low-resource countries such as China and can serve to allow scalable resource access to MDD care for low- and middle-income countries.

## Appendix

Multimedia Appendix 1 Outline of the treatment content, homework, and corresponding screenshots of the internet-based cognitive behavioral therapy program.

Multimedia Appendix 2 Means and SEs for utility measured using the Short-Form Six-Dimension health index and resulting quality-adjusted life year values by trial arm and time point in the intention-to-treat and scenario analyses.

Multimedia Appendix 3 Means and SEs for costs by trial arm and time point in the intention-to-treat and scenario analyses.

Multimedia Appendix 4 Regression-adjusted costs and quality-adjusted life year estimates by trial arm and difference in means between trial arms from the societal perspective.

Multimedia Appendix 5 Regression-adjusted costs and quality-adjusted life year estimates by trial arm and difference in means between trial arms from the health care system perspective.

Multimedia Appendix 6 Consolidated Health Economic Evaluation Reporting Standards 2022 checklist.

Multimedia Appendix 7 CONSORT-eHEALTH checklist (V 1.6.1).

## Abbreviations

CC: complete case
CEAC: cost-effectiveness acceptability curve
CONSORT-EHEALTH: Consolidated Standards of Reporting Trials of Electronic and Mobile Health Applications and Online Telehealth
CUA: cost utility analysis
HRQoL: health-related quality of life
ICBT: internet-based cognitive behavioral therapy
ICUR: incremental cost utility ratio
ITT: intention to treat
MDD: major depressive disorder
PHQ-9: Patient Health Questionnaire–9
QALY: quality-adjusted life year
RCT: randomized controlled trial
SDS: Sheehan Disability Scale
SF-6D: Short-Form Six-Dimension health index
WTP: willingness to pay

## Data Availability

The datasets generated and analyzed during this study are available from the corresponding author on reasonable request.
