# Supplementary material for: Cost Utility Analysis of Internet-Based Cognitive Behavioral Therapy for Major Depressive Disorder: Randomized Controlled Trial
Source: J Med Internet Res. 2025 Feb 19;27:e67567. doi: 10.2196/67567 (PMC11888078; doi:10.2196/67567)

**Multimedia Appendix 1.** Outline of the treatment content, homework and corresponding screenshots of the internet-based cognitive behavioral therapy program.

| **Module** | **Treatment Theme** | **Content** | **Duration** | **Homework** |
| --- | --- | --- | --- | --- |
| 1 | Psychoeducation | Psychoeducation about depression, CBT^a^, the thoughts-feelings-actions cycle and events record | 36 mins | Pleasant events record |
| 2 | Recording activities and identifying your depression | Strengthening the concept of CBT triad; learning how to set goals, record activities and automatic thought; identifying specific maladaptive thoughts and behavior patterns | 24 mins | Goals setting |
|  |  |  |  | Activities record |
|  |  |  |  | Automatic thoughts record |
| 3 | Behavioral activation and identifying your thinking traps | Learning how to schedule activities and increase level of activity reasonably; introduction of common thinking traps of depression | 33 mins | Activities treasure chest |
|  |  |  |  | Activities scheduling |
|  |  |  |  | Activities record |
|  |  |  |  | Automatic thoughts record |
| 4 | Procrastination and thought challenging | Task decomposition to change avoidance behavior and procrastination; adjusting and challenging negative automatic thoughts with "thought-monitor table" | 25 mins | Task decomposition |
|  |  |  |  | Activities scheduling |
|  |  |  |  | Activities record |
|  |  |  |  | Automatic thoughts record |
| 5 | Attribution training and problem solving | Strategies about rational attribution and problem solving | 26 mins | Attribution training |
|  |  |  |  | Activities scheduling |
|  |  |  |  | Activities record |
|  |  |  |  | Problem solving |
| 6 | Realistic thinking | Identifying and evaluating your inner conviction; making more adaptive response to yourself, others and the world | 23 mins | Activities scheduling |
|  |  |  |  | Activities record |
|  |  |  |  | Identifying inner conviction |
|  |  |  |  | Evaluating inner conviction |
| 7 | Maintaining the effect and relapse prevention | Summaries and reviews about challenging depression; strategies about relapse prevention | 20 mins | New goals |

^a^CBT: cognitive behavioral therapy.


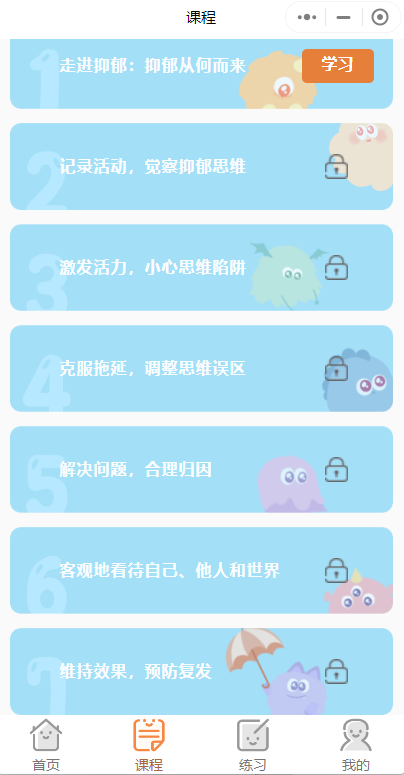


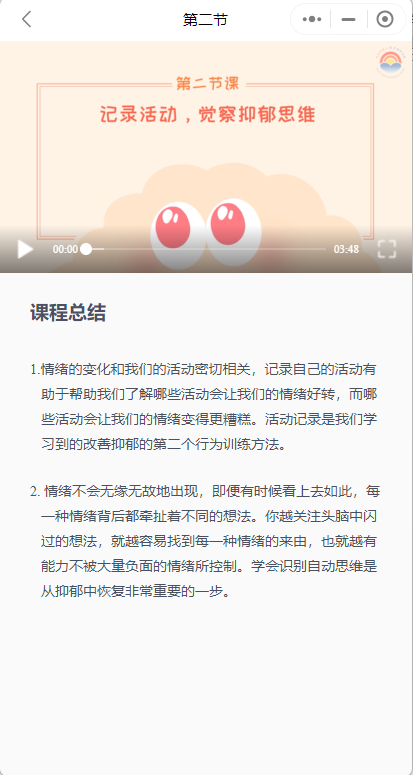

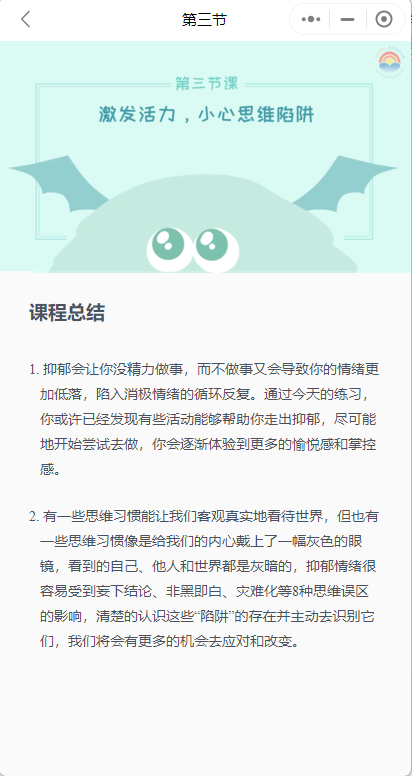

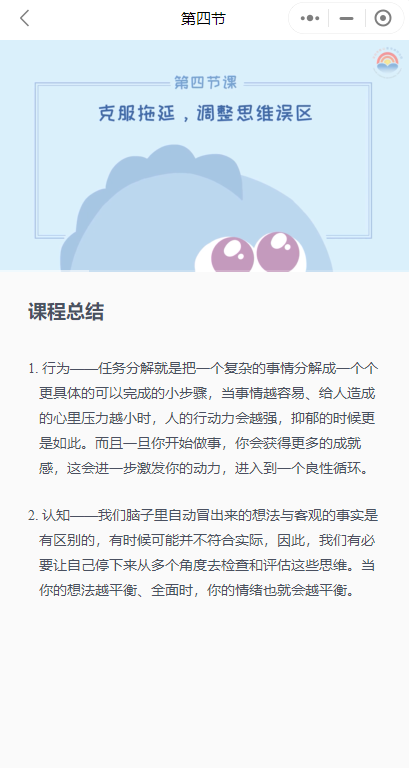

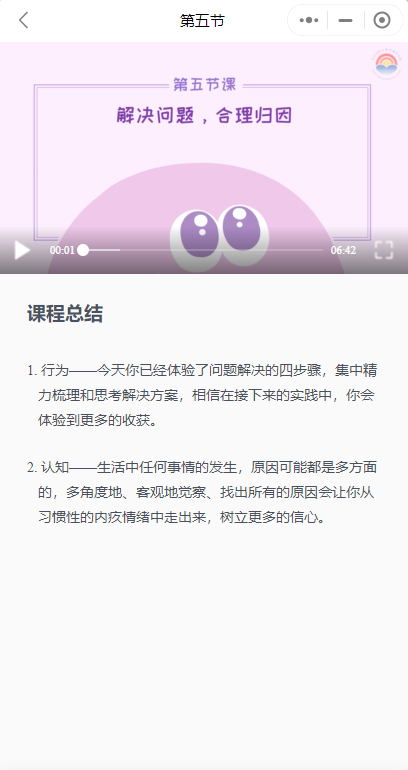

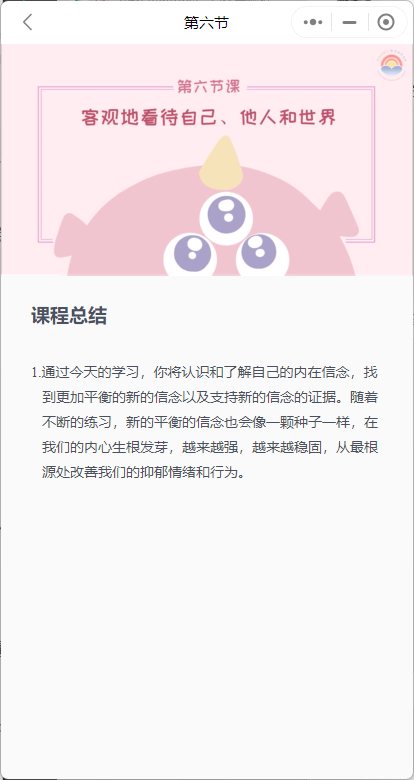

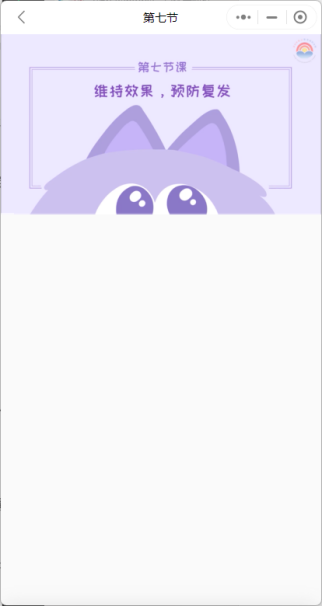

Supplement: Multimedia Appendix 1 [file jmir_v27i1e67567_app1.docx]
