# Supplementary material for: Cost Utility Analysis of Internet-Based Cognitive Behavioral Therapy for Major Depressive Disorder: Randomized Controlled Trial
Source: J Med Internet Res. 2025 Feb 19;27:e67567. doi: 10.2196/67567 (PMC11888078; doi:10.2196/67567)
Supplement: Multimedia Appendix 2 [file jmir_v27i1e67567_app2.docx]

**Multimedia Appendix 2. Mean and SEs for utility measured using the Short-Form Six-Dimension health index and resulting quality-adjusted life year values by trial arm and time point in the intention-to-treat and scenario analyses**

| **Time points** | **ICBT^a^** | | **Waitlist control** | | **Dif. Mean** | |
| --- | --- | --- | --- | --- | --- | --- |
|  | **Utility, Mean (SE)** | **QALY, Mean (SE)** | **Utility, Mean (SE)^b^** | **QALY, Mean (SE)** | **Utility,**  **Mean (*P* value)^c^** | **QALY,**  **Mean (*P* value)^c^** |
| Baseline | 0.5190 (0.0092) |  | 0.5625 (0.0079) |  | -0.0435 (0.11) |  |
| 8 weeks | 0.6121 (0.0089) | 0.1414 (0.0021) | 0.6075 (0.0087) | 0.1463 (0.0019) | -0.0046 (0.91) | -0.0049 (0.41) |
| 3 months | 0.6187 (0.0093) | 0.2952 (0.0041) | 0.6222 (0.0071) | 0.3000 (0.0038) | -0.0035 (0.72) | -0.0047 (0.67) |
| 6 months | 0.6504 (0.0086) | 0.4539 (0.0059) | 0.6562 (0.0050) | 0.4598 (0.0053) | -0.0058 (0.84) | -0.0059 (0.73) |
| 12 months | 0.6400 (0.0102) | 0.7765 (0.0096) | 0.6404 (0.0059) | 0.7839 (0.0080) | -0.0004 (0.42) | -0.0074 (0.72) |
| ^a^ICBT, Internet-Based Cognitive Behavioral Therapy;  ^b^No data was collected for the waitlist control group beyond 8 weeks; The values presented for the waitlist control group and difference in means beyond 8 weeks were based on the scenario analyses and were presented here for descriptive purpose only to be cross-referenced with the QALY values presented in the scenario analyses;  ^c^Utility and QALY at different time points were compared between the 2 groups using t tests. | | | | | | |
