# Supplementary material for: Cost Utility Analysis of Internet-Based Cognitive Behavioral Therapy for Major Depressive Disorder: Randomized Controlled Trial
Source: J Med Internet Res. 2025 Feb 19;27:e67567. doi: 10.2196/67567 (PMC11888078; doi:10.2196/67567)
Supplement: Multimedia Appendix 3 [file jmir_v27i1e67567_app3.docx]

**Multimedia Appendix 3.** **Mean and SEs for costs by trial arm and time point in the intention-to-treat and scenario analyses**

| **Time points** | **ICBT^a^ (n=122),  costs (CNY^b^)** | **Waitlist**  **control (n=122),  costs (CNY)^c^** | ***P* value^d^** |
| --- | --- | --- | --- |
|  | Mean (SE) | Mean (SE) |  |
| **Baseline** |  |  |  |
| Direct medical costs | 10243.1 (667.2) | 8622.1 (458.3) | 0.317 |
| Indirect costs | 22083.8 (648.9) | 18853.1 (653.7) | 0.106 |
| **8 weeks** |  |  |  |
| Direct medical costs | 2421.2 (501.1) | 1417.9 (189.6) | 0.250 |
| Indirect costs | 14601.8 (434.1) | 10516.5 (447.9) | 0.001 |
| **3 months** |  |  |  |
| Direct medical costs | 537.4 (139.9) | 360.8 (43.6) | <0.001 |
| Indirect costs | 8156.5 (626.8) | 6937.8 (133.8) | <0.001 |
| **6 months** |  |  |  |
| Direct medical costs | 366.6 (74.6) | 320.1 (8.8) | <0.001 |
| Indirect costs | 10874.4 (693.5) | 9333.9 (213.6) | <0.001 |
| **12 months** |  |  |  |
| Direct medical costs | 791 (109.7) | 722.2 (14.2) | <0.001 |
| Indirect costs | 14434.1 (1040.7) | 12949.1 (192.9) | <0.001 |
| ^a^ICBT, Internet-Based Cognitive Behavioral Therapy;  ^b^CNY, Chinese Yuan;  ^c^No data was collected for the waitlist control group beyond 8 weeks; The values presented for the waitlist control group and the difference in means beyond 8 weeks are based on the scenario analyses and are presented here for descriptive purposes only to be cross-referenced with the cost values presented in the scenario analyses;  ^d^Costs at different time points were compared between the 2 groups using Mann-Whitney *U* tests. | | | |
