# Supplementary material for: Cost Utility Analysis of Internet-Based Cognitive Behavioral Therapy for Major Depressive Disorder: Randomized Controlled Trial
Source: J Med Internet Res. 2025 Feb 19;27:e67567. doi: 10.2196/67567 (PMC11888078; doi:10.2196/67567)
Supplement: Multimedia Appendix 4 [file jmir_v27i1e67567_app4.docx]

| **Multimedia Appendix 4. Regression-adjusted costs and quality-adjusted life year estimates by trial arm and difference in means between trial arms from the societal perspective** | | | | | | | | | | | |
| --- | --- | --- | --- | --- | --- | --- | --- | --- | --- | --- | --- |
| **Analysis** | **ICBT^a^** | | | | **Waitlist control** | | | | **Dif. Means^b^** | | |
|  | **%** | **Mean** | **bSE^c^** | **95%*CI*** | **%** | **Mean** | **bSE** | **95%*CI*** | **Mean** | **bSE** | **95%*CI*** |
| **Complete case (CC)-8 weeks** | | | | | | | | | | | |
| BA^d^ costs (CNY^e^) | 71.3 | 12986.65 | 1198.87 | (10636.91,15336.38) | 94.3 | 13136.29 | 1085.00 | (11009.74,15262.85) | -149.65 | 1645.32 | (-3264.72,3069.91) |
| BA QALYs | 71.3 | 0.1448 | 0.0022 | (0.1405,0.1490) | 94.3 | 0.1405 | 0.0018 | (0.1370,0.1440) | 0.0042 | 0.0028 | (-0.0013,0.0098) |
| **Intention-to-treatment (ITT)-8 weeks** | | | | | | | | | | | |
| BA costs (CNY) | 100.0 | 16845.84 | 1033.40 | (14820.49,18871.2) | 100.0 | 17745.29 | 1676.53 | (14459.41,21031.17) | -899.45 | 2064.49 | (-4982.08,2463.78) |
| BA QALYs | 100.0 | 0.1461 | 0.0019 | (0.1424,0.1499) | 100.0 | 0.1415 | 0.0020 | (0.1376,0.1454) | 0.0046 | 0.0028 | (-0.0008,0.0101) |
| **Intention-to-treatment (ITT)-3 months^f^** | | | | | | | | | | | |
| BA costs (CNY) | 100.0 | 25349.96 | 2011.71 | (21407.09,29292.84) | 100.0 | 26628.89 | 2595.26 | (21542.2811,31715.5069) | -1278.93 | 3378.79 | (-7799.37,4632.33) |
| BA QALYs | 100.0 | 0.3039 | 0.0057 | (0.2927,0.3151) | 100.0 | 0.2913 | 0.0056 | (0.2803,0.3022) | 0.0126 | 0.0079 | (-0.0028,0.0281) |
| **Intention-to-treatment (ITT)-6 months^f^** | | | | | | | | | | | |
| BA costs (CNY) | 100.0 | 36349.81 | 3036.76 | (30398.03,42301.59) | 100.0 | 38111.04 | 3750.90 | (30759.426,45462.55) | -1761.24 | 4952.93 | (-11171.09,7214.37) |
| BA QALYs | 100.0 | 0.4658 | 0.0089 | (0.4483,0.4833) | 100.0 | 0.4478 | 0.0087 | (0.4307,0.465) | 0.0179 | 0.0116 | (-0.0048,0.0407) |
| **Intention-to-treatment (ITT)-12 months^f^** | | | | | | | | | | | |
| BA costs (CNY) | 100.0 | 51106.12 | 4626.22 | (42039.76,60172.48) | 100.0 | 53805.06 | 5503.65 | (43018.1171,64591.9949) | -2698.94 | 7396.60 | (-16649.71,10782.51) |
| BA QALYs | 100.0 | 0.7941 | 0.0172 | (0.7604,0.8278) | 100.0 | 0.7663 | 0.0156 | (0.7356,0.7969) | 0.0278 | 0.0211 | (-0.0136,0.0692) |
| ^a^ICBT, Internet-Based Cognitive Behavioral Therapy;  ^b^Dif. Mean, the difference in mean values between trial arms;  ^c^bSE, Bootstrapped Standard Error;  ^d^BA, baseline adjusted;  ^e^CNY, Chinese Yuan;  ^f^ The scenario analyses represent a scenario whereby the waitlist control participants' health-related quality of life and care costs after 8 weeks up to twelve months followed the same trend as was observed in the intervention group; therefore, the difference in costs and QALYs are based on the observed values for the intervention group at each time-point but predicted values using regression analysis for the waitlist control, the regression model for which is described in the methods section. Incremental results from the societal perspective are the ‘ICBT intervention’ group (CC N =86; ITT N =122) minus the ‘waitlist control’ group (CC N =115; ITT N = 122). | | | | | | | | | | | |
